# *De novo* transcriptome of *Ischnura elegans* provides insights into sensory biology, colour and vision genes

**DOI:** 10.1186/1471-2164-15-808

**Published:** 2014-09-22

**Authors:** Pallavi Chauhan, Bengt Hansson, Ken Kraaijeveld, Peter de Knijff, Erik I Svensson, Maren Wellenreuther

**Affiliations:** Department of Biology, Lund University, Sölvegatan 37, SE 22362 Lund, Sweden; Animal Ecology, Department of Ecological Science, VU University Amsterdam, De Boelelaan 1085, 1081 HV Amsterdam, The Netherlands; Department of Human and Clinical Genetics, Leiden University Medical Center, PO Box 9600, 2300 RC Leiden, The Netherlands

**Keywords:** Odonata, Zygoptera, Polymorphisms, Transcriptome assembly, RNA-seq, Opsin, Melanin, Ommochrome and pteridine, Thermal adaptation

## Abstract

**Background:**

There is growing interest in odonates (damselflies and dragonflies) as model organisms in ecology and evolutionary biology but the development of genomic resources has been slow. So far only one draft genome (*Ladona fulva*) and one transcriptome assembly (*Enallagma hageni*) have been published. Odonates have some of the most advanced visual systems among insects and several species are colour polymorphic, and genomic and transcriptomic data would allow studying the genomic architecture of these interesting traits and make detailed comparative studies between related species possible. Here, we present a comprehensive *de novo* transcriptome assembly for the blue-tailed damselfly *Ischnura elegans* (Odonata: Coenagrionidae) built from short-read RNA-seq data. The transcriptome analysis in this paper provides a first step towards identifying genes and pathways underlying the visual and colour systems in this insect group.

**Results:**

Illumina RNA sequencing performed on tissues from the head, thorax and abdomen generated 428,744,100 paired-ends reads amounting to 110 Gb of sequence data, which was assembled *de novo* with Trinity. A transcriptome was produced after filtering and quality checking yielding a final set of 60,232 high quality transcripts for analysis. CEGMA software identified 247 out of 248 ultra-conserved core proteins as ‘complete’ in the transcriptome assembly, yielding a completeness of 99.6%. BLASTX and InterProScan annotated 55% of the assembled transcripts and showed that the three tissue types differed both qualitatively and quantitatively in *I. elegans*. Differential expression identified 8,625 transcripts to be differentially expressed in head, thorax and abdomen. Targeted analyses of vision and colour functional pathways identified the presence of four different opsin types and three pigmentation pathways. We also identified transcripts involved in temperature sensitivity, thermoregulation and olfaction. All these traits and their associated transcripts are of considerable ecological and evolutionary interest for this and other insect orders.

**Conclusions:**

Our work presents a comprehensive transcriptome resource for the ancient insect order Odonata and provides insight into their biology and physiology. The transcriptomic resource can provide a foundation for future investigations into this diverse group, including the evolution of colour, vision, olfaction and thermal adaptation.

**Electronic supplementary material:**

The online version of this article (doi:10.1186/1471-2164-15-808) contains supplementary material, which is available to authorized users.

## Background

Odonata display large inter- and intra-specific colour variation and have some of the most advanced visual systems among insects [1, 2]. With their large and complex eyes, aquatic and terrestrial life stages [3], carnivorous lifestyle [4], exceptional mating behaviours [5, 6], diversity in coloration [7], and occupancy of diverse light environments, odonates are ideal model organisms to study the evolution of colour and vision pathways and functions. However, odonate colour and visual systems are little understood [8]. Lack of genomic and transcriptomic sequence information limits molecular investigation on this group. So far only one draft genome (BioProject PRJNA194433, *Ladona fulva*) and one transcriptome assembly [9] have been published for odonates. An improved understanding of the molecular basis of phenotypic adaptations in Odonata would allow investigations of genomic divergence associated with ecological shifts in light environments, and inter- and intra-specific divergence in color vision. Several distinctive traits of the blue-tailed damselfly *Ischnura elegans* (Odonata: Coenagrionidae) make this species a useful model for studying genome evolution and development. *Ischnura elegans* has developed into a model organism in evolutionary ecology because of its female limited colour polymorphism, which affects mate choice and sexual conflict interactions. Males of *I. elegans* are monomorphic in colour, but females of this species fall into one of three distinct phenotypically visible colour morphs, namely the male mimicking androchrome morph, and the more cryptic infuscans and infuscans-obsoleta morph [10]. The prevalence of female colour polymorphism in this species is thought to result from sexual conflict over optimal mating rates, where females might benefit from lower mating rates than males, and where pre-copulatory male mating harassment is common [11, 12]. This sexual conflict leads to extensive mating harassment and negative frequency-dependent selection, because the males form search images for the common morphs, similar to the apostatic survival selection on common prey caused by predators [13]. This species has also been studied with respect to sperm competition [14, 15], morph dependent mating rates [11, 12], and the evolution of reproductive barriers [16, 17]. *Ischnura elegans* belongs to the largest damselfly family Coenagrionidae, which includes 95 genera and 1082 species worldwide [18]. Over 100 species are colour polymorphic [19], and evidence from crossing experiments in several species suggests a genetic basis to colour [20, 21]. In the female-polymorphic genus *Ischnura*, even closely related taxa often differ in the presence and absence of female polymorphism and/or in the spectral ability to differentiate colour [22]. Identifying the genetic changes underlying the colour polymorphism on an intra- and inter-specific level would increase our understanding of the macroevolutionary dynamics of this polymorphism. *Ischnura elegans* is a widespread damselfly species all over Europe [23, 24] and can commonly be found in disturbed environments, such as human-made artificial ponds [13]. Unlike many other odonate species, *I. elegans* tolerates most plants as perching substrate [3].

Here we present a *de novo* transcriptome assembly for the blue-tailed damselfly *I. elegans* to investigate the nuclear, protein-encoding gene profile of this species and to give functional annotation to the proteins expressed. The transcriptome of the head, thorax and abdomen are compared to each other, and to the transcriptome of the dragonfly *Ladona fulva* (Odonata: Anisoptera) [BCM-HGSC:I5K] [25], the damselfly *Enallagma hageni* (Odonata: Zygoptera) [NCBI:SRR649536] [9, 26] and the fruit fly *Drosophila melan*o*gaster* (Diptera) [Ensembl:BDGP5] [27]. Furthermore, we aim to generate a sensory toolkit for the genes underlying colour recognition (e.g. opsins), female polychromatism and body colour patterns (e.g. melanin pathway).

## Results and discussion

### Transcriptome sequencing

Illumina sequencing of one *I. elegans* individual yielded a total of 110 Gb of mRNA sequence equivalents consisting of 428,744,100 paired-ends 100 bp reads (155,232,504 reads from the head, 159,734,116 from the thorax and 113,777,480 from the abdomen, respectively). The average read length for each of the three tissues was 99 bp, yielding complete datasets of 39.8 Gb for the head, 40.8 Gb thorax and 29.2 Gb for the abdomen. Quality parameters of the three tissues types (head, thorax and abdomen) were 91%, 92%, 92% for Q20, and 42%, 38%, and 43% for the GC percentage, respectively, while the percentage of unknown base calls (N) was 0.007% for both the head and thorax and 0.005% for the abdomen.

High quality data (clean reads) were obtained by removing reads containing adapter sequences, short reads and low quality reads from raw sequence data, reducing the three sequence sets to approximately 129, 134, and 95 million reads for the head, thorax and abdomen, respectively. Subsequently, the trimmed reads from the head, thorax and abdomen had a mean read length of 83, 84 and 82 bp, respectively, and the Q20 percentage was 100% for all three tissues. For detail summary of the trimming step statistics refer to Table 1. All the subsequent analyses were carried out using 357,641,660 high quality trimmed reads.

**Table 1:** **Trimming report**

	Head	Thorax	Abdomen
Number of reads before trimming	155232504	159734116	113777480
Reads kept after trimming	128679780	133566318	95395562
Percentage of reads discarded	17.1%	16.4%	16.2%
Reads average length before trimming	99	99	99
Reads average length after trimming	83	84	82
Q20% before trimming	91%	92%	92%
Q20% after trimming	100%	100%	100%
Q30% before trimming	83%	84%	83%
Q30% after trimming	96%	96%	96%
Total high quality reads	357641660		

### *De novo*transcriptome assembly, quality filtering and assessment

The transcriptome was assembled *de novo* with Trinity [28, 29] using all trimmed reads and yielded a total of 89,708 contigs with a minimum length of 201 bp, a N50 value of 2,610 bp and an average contig length of 1,213 bp. In the absence of a reference genome it is difficult to assess the quality of the assembled transcripts. However, to identify poor quality and potentially mis-assembled transcripts, the reads were mapped back to the assembly and the alignment visualized with IGV v.2.3.2 [30].

To improve the overall quality of the assembled transcriptome, a three-step quality filtering method was employed [31–33]. First, sequence redundancy was removed by clustering the duplicates using CDHIT-EST at 95% sequence similarity [34]. This step clustered 14.8% of the transcripts together, leaving 76,356 transcripts. Second, the transcript read coverage at each base was calculated using BED Tools. Transcripts that had a mean coverage per base of less than 5 were removed, filtering 16,008 transcripts, and leaving 60,348 high quality transcripts. Third, RepeatMasker [35] identified 17,467 repetitive elements, 178 RNA (tRNA, rRNA and srpRNA) and 2,691 low complexity regions in the transcriptome (Additional file 1: Table S1). From this, 138 ribosomal RNA sequences were identified in 116 transcripts (0.2%) and these were removed from the assembly. The filtering step not only reduced the redundancy but also filtered the shorter sequences (Additional file 2: Figure S1). Comparative assembly statistics before and after filtering are reported in Additional file 1: Table S2. The final dataset contained 60,232 high quality transcripts with an N50 value of 2,571 bp and a mean length of 1,281 bp, which was used for all subsequent analyses. A detailed summary of the final assembly statistics can be found in Table 2.

**Table 2:** **Summary statistics of final assembly**

Assembly assessment parameters	Final transcript set
Number of contigs	60232
Total size of contigs (bp)	77140699
Longest contig (bp)	24097
Shortest contig (bp)	201
Mean contig size (bp)	1281
Median contig size (bp)	627
N50 contig length (bp)	2571
Number of contig > 500 nt	34746
Number of contig > 1000 nt	22164

After quality filtering, the assembly was further validated for sequence completeness. CEGMA [36] identified 247 out of 248 ultra-conserved core proteins as ‘complete’ in the transcriptome assembly, yielding a completeness of 99.6%. The remaining gene was identified as a ‘partial’ gene. TargetIdentifier [37] identified 23,021 transcripts with a BLASTX hit, of which 15,949 transcripts (69%) could be assembled to their full length. Of these transcripts, 14,301 were identified as full-length, 1,496 as short full-length, 152 as ambiguous, 3,983 as 5′-sequenced partial and 3,089 as 3′-sequenced partial. The full-length information was generated only for the transcripts that yielded a BLASTX hit. Further, the assembly was investigated for the ability to yield protein-coding sequences. TransDecoder reported 24,885 ORFs in 21,317 (35.4%) transcripts. The assembly sequence completeness and protein-yielding capability was high, and hence the assembly was used for further analysis.

### Transcript annotation

**Figure 1:**
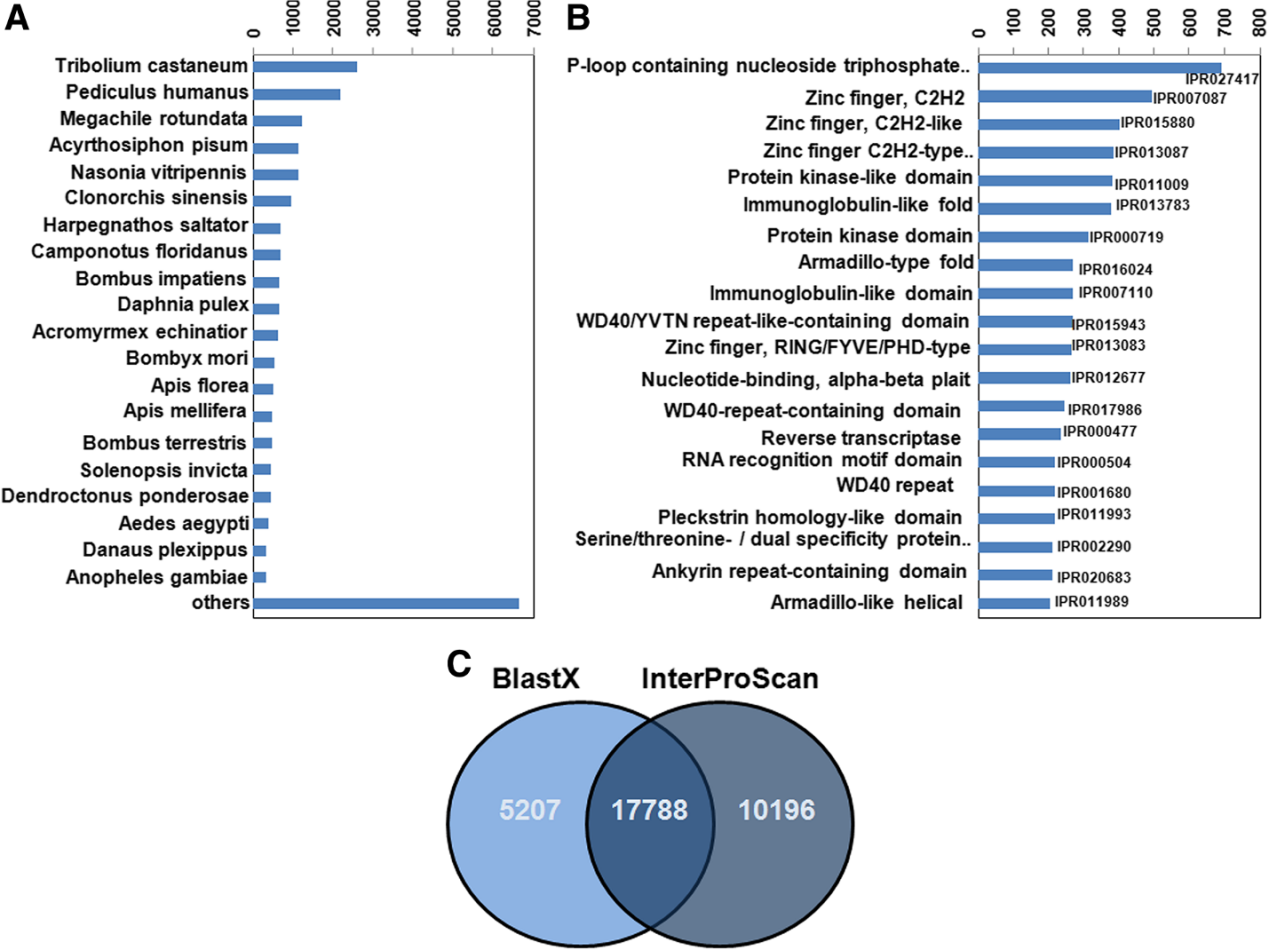
**Transcriptome annotation. A)** Top 20 BLASTX species hits. **B)** 20 most abundant InterPro domains identified in *I. elegans*: the right side of the histogram shows the IPR numbers (InterProScan accession number) while the left side of the histogram explains them. **C)** Overlap of BLASTX (light blue) and InterProScan (dark blue) annoted transcripts.

The transcript annotation with BLASTX [38] using the NCBI non-redundant (nr) protein database gave a match for 22,995 (38.2%) transcripts, while 37,237 transcripts could not be matched with BLASTX using a cut-off of 1E-5. The species representing the top BLAST hits are shown in Figure 1A. Of these, *Tribolium castaneum* (Coleoptera) yielded the highest number of top hits, followed by *Pediculus humanus* (Phthiraptera) and *Megachile rotundata* (Hymenoptera). All three orders belong to the Neoptera reviewed in [39], which is considered the closest relative to the Odonata (Metapterygota). Of the three orders, the Phthiraptera are taxonomically closest to the Odonata, followed by the Hymenoptera and then the Coleoptera [39, 40], which was not reflected in our BLASTX homology search. The small genome size of Phthiraptera and its high evolutionary rate (long branch length [40]), likely due to the parasitic lifestyle, possibly partly explains the lower number of hits in comparison to Coleoptera. Moreover, all the three orders diverged from Odonates a long time ago and during a relatively small time window, making a distinction between them difficult. Lastly, the homology results will likely be biased by the amount and quality of available species-specific data that has been deposited in the databases. Closely related species with genomic resources were compared to the *I. elegans* transcriptome by mapping our data to the transcriptome of the dragonfly species *Ladona fulva* (Odonata), the damselfly species *Enallagma hageni* (Odonata) and the fruit fly model *Drosophila melanogaster* (Diptera).

The BLAST2GOInterProScan annotation resulted in 27,984 transcripts (46.5%) with at least one InterProScan annotation. A list of the 20 most abundant InterPro domain hits is reported in the Figure 1B, showing IPR027417 (P-loop containing nucleoside triphosphate hydrolase) to be the most prevalent domain present in 691 transcripts, followed by IPR007087 (Zinc finger, C2H2) and IPR015880 (Zinc finger, C2H2 like). The assembled transcripts were also annotated with Gene Ontology (GO) into three major GO categories: Biological Processes, Cell Component, and Molecular Function. A total of 11,748 (20%) transcripts were associated with at least one GO term: 6,393 transcripts were assigned to the Biological Processes, 10,483 to the Molecular Function and 3,848 to Cell Components (Additional file 2: Figures S2, S3 and S4). In the Biological Process category, the majority of transcripts were involved in cellular protein metabolic processes (GO: 0006464) and signal transduction processes (GO: 0007165). A large fraction of transcripts in the Molecular Function category is involved in DNA binding (GO: 0003677) and RNA binding (GO: 0003723) functions, whereas the Cellular Component category is predominated by transcripts involved in intracellular organelle (GO: 0043229) and cytoplasm (GO: 0005737) processes.

BLASTX was able to annotate 38.2% and InterProScan 46.5% of the transcripts. Considering both the BLASTX and InterProScan results, a total of 33,191 (55.1%) *de novo* assembled transcripts could be annotated. Of the total number of annotated transcripts, 17,788 transcripts were well annotated (assigned with a gene name as well as a protein signature), obtained from union of BLASTX and InterProScan annotated transcripts, detailed in Figure 1C.

### RNA-sequence mapping on the *D. melanogaster*and *I. elegans*transcriptome

Almost all *I. elegans* trimmed sequence reads (99.4% from all tissues) failed to map to the *D. melanogaster* transcriptome, which consists of 27,142 transcripts [27]. Of the 2,283,100 reads (0.6%) that mapped to the *D. melanogaster* transcriptome, 721,518 reads (0.2%) were paired, whereas 1,561,582 reads (0.4%) mapped as singletons. Only 1,626 (6.0%) of the *D. melanogaster* transcripts showed expression in *I. elegans* (a list of the 30 most expressed transcripts are shown in Additional file 1: Table S3).

RNA-seq reads from the dragonfly *L. fulva* [25] and the damselfly *E. hageni* [26] were mapped to the *I. elegans* transcriptome. The majority of reads (88.9%) from the *L. fulva* could not be mapped to the *I. elegans* transcriptome. Among the 8,569,657 *L. fulva* reads that mapped to the *I. elegans* transcriptome, 4,565,885 (5.9%) mapped as singletons and 4,003,772 (5.2%) reads as pairs, generating an overall mapping percentage of 11.1%. Mapping of *E. hageni* single-end reads to the *I. elegans* transcriptome resulted in 513,478 (52.6%) reads that could not be mapped and 463,268 (47.4%) of reads mapped. Summary statistics of RNA-seq reads for *L. fulva* and *E. hageni* mapping to the *I. elegans* transcriptome are reported in Table 3. More *E. hageni* (11.6%) transcripts were found in *I. elegans* compared to *L. fulva* (3.7%)*.* Of these, 1,325 (2.2%) transcripts were expressed in both the species (Additional file 2: Figure S5). They are presumably expressed in all three species, but homology is too low to detect most of them. A list of the 30 most expressed genes from both species is shown in Additional file 1: Table S4.

**Table 3:** **Statistics for RNA-seq mapping on** ***I. elegans*** **transcriptome**

Mapping parameters	***Ladona fulva***	***Enallagma hageni***
Total reads	77135056	976765
Reads mapped in pairs	4003772	-
Reads mapped in broken pairs	4565885	463268
Percentage of mapped reads	11.11%	47.43%
Reads not mapped	68565399	513497

Interspecific transcript level comparisons showed that *E. hageni* shared most transcripts (6,989) with *I. elegans*, followed by *L. fulva* (2,244) and *D. melanogaster* (1,626), which closely corresponds to the taxonomic distance between these species [39, 40]. A large fraction of these common transcripts encodes for proteins that are involved in maintaining structure and function of muscles and the transfer of electrons in the electron transport chain in mitochondria.

### Abundance estimation and differential expression of transcripts in the three tissue types

**Figure 2:**
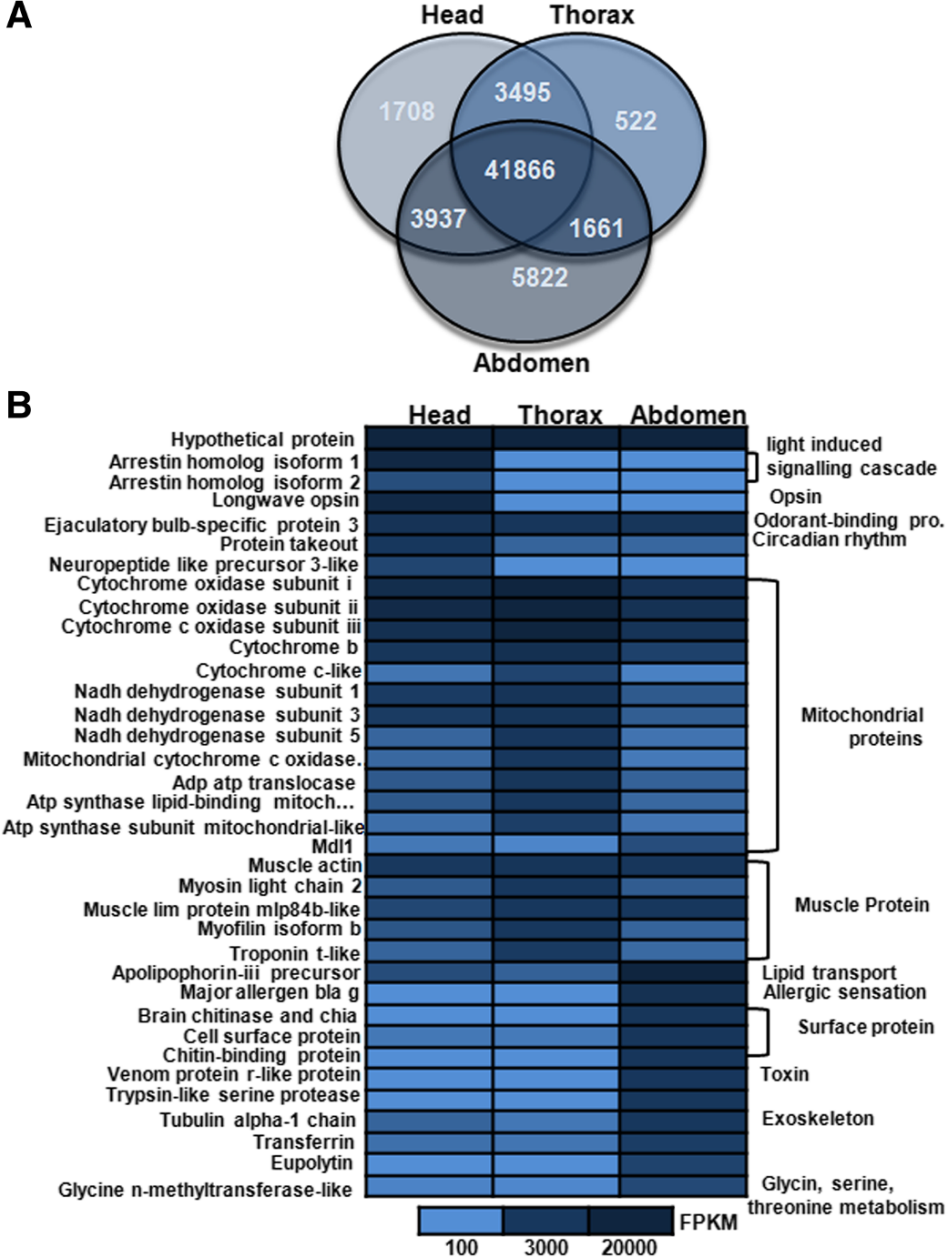
**Summary of transcriptome expression analysis. A)** Overlap of the head, thorax and abdomen transcriptome. The abdomen transcriptome is larger than the two other tissue types, with 53,286 transcripts detected in the abdomen (dark blue), 51,006 in the head (light blue) and 47,544 in the thorax (blue). **B)** Heatmap of RNA abundance expression. Abundance expression was determined using RSEM and abdundance was measured using FPKM (Fragments Per Kilo base of transcript per Million mapped reads). The figure depicts the 20 most abundantly expressed genes from the head, thorax and abdomen, supporting data is given in the Additional file 1: Tables S5, S6 and S7. The left side of the figure represents the gene name, while the right side describes the general function of the gene.

RNA-seq mapping was performed with RSEM [41] to calculate expression levels of the assembled transcripts for the three tissue types. Mapping results showed that 54,856,781 (85.3%) paired reads from the head, 55,385,379 (83.0%) from the thorax and 39,821,676 (83.5%) from the abdomen mapped to the assembled transcript, resulting in more than 15% reads that could not be mapped to the final set of assembled transcripts. The RNA-seq mapping analysis revealed that more transcripts were expressed in the abdomen tissue than in the other two tissue types. The total number of transcripts expressed was 51,006 (84.7%) in the head, 47,544 (79.0%) in the thorax and 53,286 (88.5%) in the abdomen, respectively. Comparative analyses among all the three tissues revealed that 41,866 (69.5%) transcripts were expressed in all tissues, while 3,495 (5.8%) of the transcripts were mutually expressed in the head and thorax, 3,937 (6.5%) in the head and abdomen and lastly 1,661 (2.8%) transcripts in the abdomen and thorax (Figure 2A).

A list of the 20 most expressed genes in the head, thorax and abdomen, respectively is reported in Figure 2B (extracted from Additional file 1: Tables S5, S6 and S7). Of the top 20 genes expressed in *I. elegans* tissues, the largest fraction was made up of proteins that act as components in the respiratory chain of mitochondria facilitating electron transfer (e.g. cytochrome b, cytochrome c, cytochrome oxidase and NADH dehydrogenase) and muscle proteins (e.g. muscle actin, muscle lib protein and myofilin). A hypothetical protein that showed highest expression in all the three tissue is an immune-related gene, which regulates immune signalling in insects [42]. Noteworthy, high expression of protein coding gene *takeout* [Flybase: FBgn0039298] was observed in head. This protein participates in a novel circadian output pathway and is also involved in male courtship behaviour in *D. melanogaster.*

**Figure 3:**
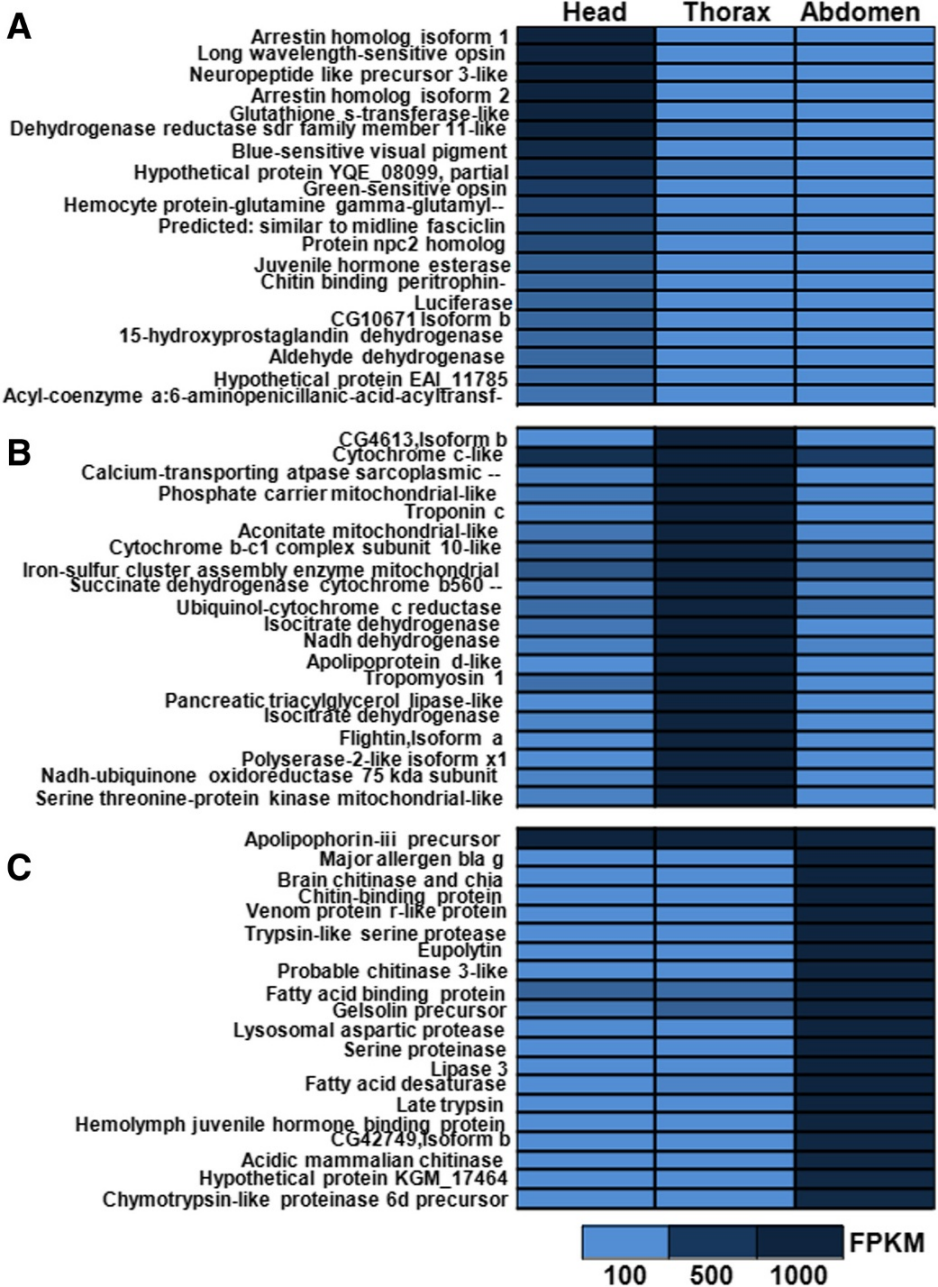
**Differential gene expression. A)** – **C)** Heatmap of RNA differential expression, head, thorax and abdomen. Expression was determined by applying differential expression analysis on final set of transcripts. The figure describes expression of 20 most differentially expressed genes from three tissue types, figure supporting data is reported in Additional file 1: Tables S8, S9 and S10.

A total of 8,625 transcripts were differentially expressed in the head (2,039), thorax (963) and abdomen (5,623) using a p-value cut-off for FDR 1E-3 and a fold change of 2. However without biological replicates, any conclusions about gene expression data can be weak. A list of the 20 most differentially expressed genes in the head, thorax and abdomen, respectively, are reported in Figure 3A-C (based on data in Additional file 1: Tables S8-S10). The results clearly showed that the most differentially expressed genes in the head were opsins (*long-wavelength-sensitive 1*, *long-wavelength-sensitive 2* and *blue-sensitive opsin*), genes that participate in light-induced cascades (e.g. *arrestin homolog isoform 1* and *2*), and neuropeptide-like precursors that influence behaviour, development, immunity and physiological processes. The thorax showed elevated expression of genes that encode mitochondrial proteins. The differentially expressed genes of the abdomen included *major allergen Bla*, which is an allergen that is responsible for respiratory disorders like asthma [43], *brain chitinase and chia*, *chitinase*, *chitin-binding protein*, all these have chitin-binding domain and form peritrophic matrix proteins of insects, *venom protein r-like*, which is a toxin and hemolymph juvenile hormone binding protein, which regulates embryogenesis, larva development and stimulates reproductive maturation.

A GO term enrichment analysis was performed on the differentially expressed genes from the head, thorax and abdomen using GOSSIP [44] Fishers Exact Test with BLAST2GO. A total of 39 enriched GO terms were identified in the head, which were subsequently reduced to 11 most specific terms. The most specific GO terms in the head included signal transduction (GO: 0007165) and responses to abiotic stimuli (GO: 0009628) under the Biological Processes category, plasma membrane (GO: 0005886) and cytoplasmic membrane-bound vesicles (GO: 00016023) under the Cell Component category and receptor activity (GO: 0004872) and ion channel activity (GO: 0005216) under the Molecular Function category. Only three enriched GO terms were observed in the thorax, which included the generation of precursor metabolites and energy (GO: 0006091) under the Biological Processes category, mitochondrion (GO: 0005739) under Cell Component category and electron transport activity (GO: 0009055) under Molecular Function category. A total of 31 enriched GO terms were observed in the abdomen, of which 15 were reduced to the most specific GO terms. Among these, the most enriched GO terms were catabolic processes (0009056) and translation (GO: 0006412) under the Biological Processes category, ribosome (GO: 0005840) and cytoskeleton (GO: 0005856) functions under the Cell Component category and structural molecular activity (GO: 0005193) and peptidase activity (GO: 0008233) under the Molecular Function category (for details refer to the Additional file 2: Figures S6-S8).

The consistent findings from the abundance estimation and differential expression analysis underscore the specific roles that these three tissue types play in *I. elegans*: the head seems to regulate not only light receptivity and vision but also other sensory processes and transmits information via electrical and chemical signals to other body parts, response to abiotic stimulus and also contain protein that can regulate male courtship behaviour; the thorax with its flight musculature has a large number of mitochondria and muscle proteins and the abdomen not only performs translation and catabolic processes but also contains some defence proteins, such as allergens and toxins.

### Opsin and pigment pathways

**Figure 4:**
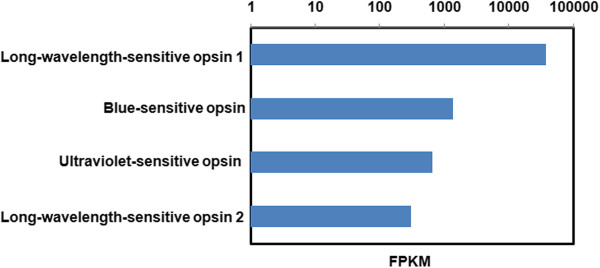
**Expression of opsins in** ***I. elegans*** **.** X-axis signifies the FPKM (Fragments Per Kilo base of transcript per Million mapped reads) value and y-axis symbolises different types of opsins identified in the transcriptome of *I. elegans*.

The odonate eye can detect colour from the ultraviolet (UV) (~300 nm) to the long wavelength (LW) (~700 nm) portion of the visible spectrum and is capable of discriminating polarized light [2, 45]. Past studies of electrophysiology have demonstrated that odonates have between 3-5 opsin copies for colour detection (reviewed in 1). We identified four types of opsins (*long-wavelength-sensitive opsin 1, long-wavelength-sensitive opsin 2, blue-sensitive opsin* and *ultraviolet-sensitive opsin*) in 20 different transcripts. The BLASTX homology search revealed that, on average, the identified opsins have more than 60% similarity with the identified homologous protein, although several showed more than 80% similarity. The abundance estimation performed on all opsins reported a FPKM (Fragments Per Kilobase of transcript per Million mapped reads) value of at least 300 in the head tissue (Additional file 1: Table S11). Among the four types of opsins detected in *I. elegans*, the long-wavelength-sensitive opsin 1 showed the highest expression in the head tissue with a FPKM of 37,939, followed by blue-sensitive opsin with a FPKM of 1,377 (Figure 4).

**Figure 5:**
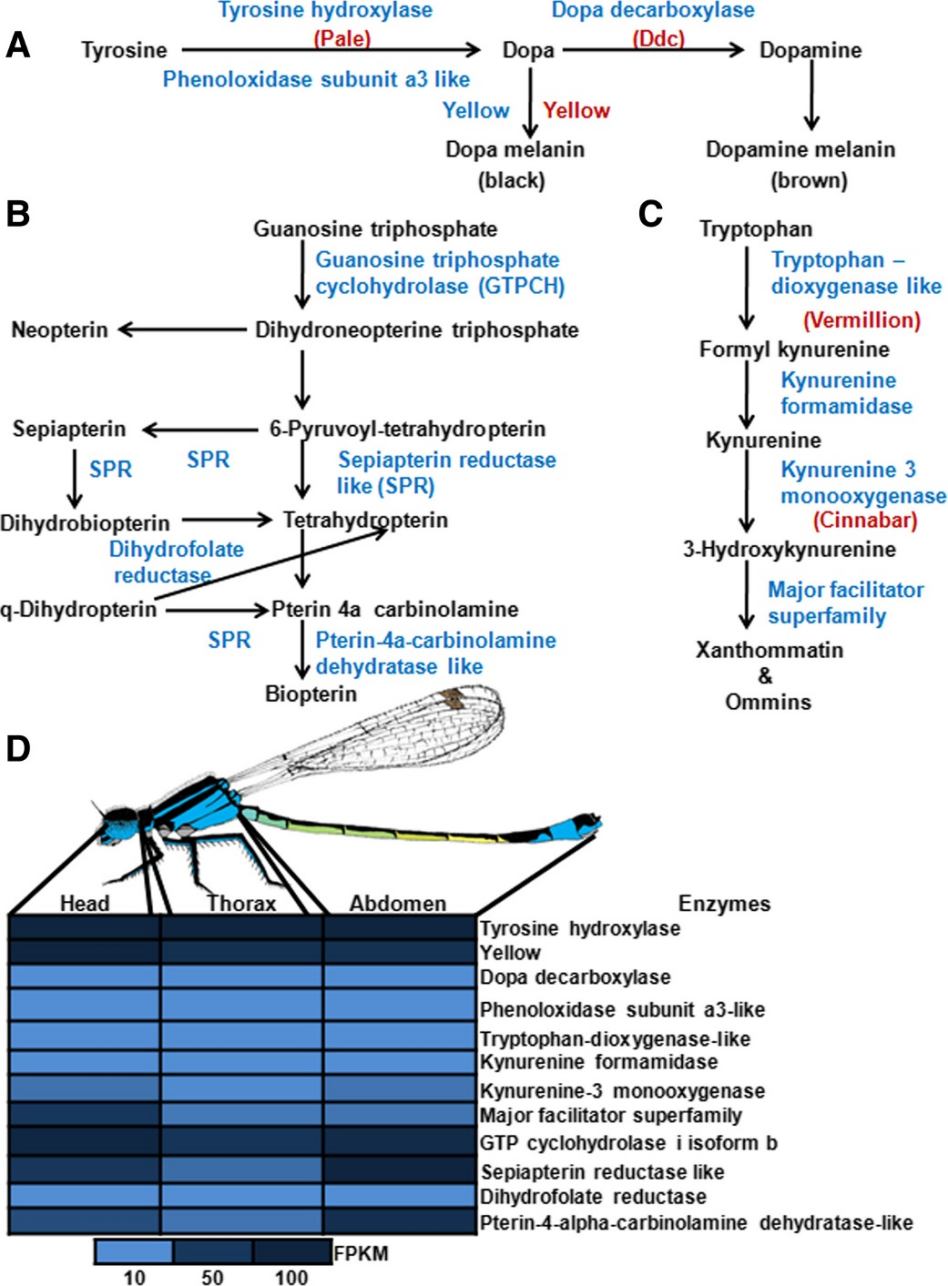
**Genes identified in** ***I. elegans*** **that are found in pigmentation pathways in experimental animal systems. A)** Melanin pathway **B)** Pteridine pathway and **C)** Ommochrome pathway. Pathways were constructed by overlying the genes identified in the *I. elegans* transcriptome over well-established colour pathways obtained from other experimental animal system. Black colour denotes pigments or pigment precursors, blue denote enzymes or proteins and red denotes genes identified in the *I. elegans* transcriptome. **D)** Heatmap of RNA expression of pigmentation enzymes in the three tissues, supporting data can be found in Additional file 1: Table S12.

In pigmentation pathways, effector genes encode for co-factors and enzymes involved in the synthesis of pigments and patterning genes regulate the distribution of pigments by influencing the activating of effector gene [46, 47]. We identified 12 enzymes in the 23 assembled transcripts belonging to three pigment pathways, namely the melanin (4), pteridine pathway (4) and ommochrome (4). The diagrammatic representation of the enzymes identified in *I. elegans* in all the three corresponding pathways is shown in Figure 5A-C. We also identified seven regulatory proteins (patterning genes) in ten transcripts.

The melanin pathway is of principal interest in insects because different components in the enzymatic pathway have different and pleiotropic effects on characters involved in mate choice, sexual selection and parasite resistance [48, 49] and possibly also learned mate preferences, which are known to occur in damselflies [50]. In the model insect *D. melanogaster*, dopamine is involved in the reward system and in learning, courtship and sexual behaviour [51–55]. The enzymes identified in *I. elegans* in the melanin pathway are tyrosine hydroxylase, dopa decarboxylase, yellow and phenoloxidases subunit a3 like, which all play important roles in forming black and brown colour pigments in other insects [46, 48, 56–58]. The phenoloxidase subunit a3 like is a copper containing oxidase that catalyses the rate-limiting conversion of tyrosine to DOPA, and DOPA to DOPA quinines [59, 60]. In calopterygid damselflies, phenoloxidase is a limiting resource in terms of the life-history allocation between sexual signalling (dark wing patches in males) and innate immune defence against parasitic infections [50]. The ommochrome pathway yields red, brown and yellow pigments. The enzymes identified in *I. elegans* in the ommochrome pathway are tryptophan 2,3-dioxygenase like, kynurenine formamidase and kynurenine 3 monooxygenase present in cell cytosol [61, 62]. The major facilitator superfamily plays an important role in the transport of 3-hydroxykynurenine into the pigment granules, where it undergoes oxidative condensation to form pigment xathommatin and ommins [63, 64]. The pteridine biosynthesis pathway produces sepiapterin and biopterin, which are yellow and blue colours. The important enzymes identified in *I. elegans* in the pathway are guanosine triphosphate cyclohydrolase, sepiapterin reductase-like, dihydrofolate reductase, pterin-4a-carbinolamine dehydratase-like [65–67]. We identified all the enzymes involved in melanin, ommochrome and pteridine pathway in *I. elegans*, except one enzyme (pyruvoyl tetrahydropterine synthase, which converts dihydroneopterine triphosphate to 6-pyruvoyl tetrahydropterine) in the pteridine pathway. The BLASTX analysis performed on all enzymes showed an average similarity of 60% with the identified homologous protein. A detailed list of all the enzymes involved in the three colour pathways with their expression in head, thorax and abdomen is shown in Figure 5D and Additional file 1: Table S12.

On the basis of RNA expression of the pigmentation enzymes reported in the Additional file 1: Table S12 and Figure 5D, the expression of tyrosine hydroxylase (FPKM = 155) and *yellow* gene (FPKM = 45) that both form black melanin is higher than dopa decarboxylase (FPKM = 1) that forms brown melanin in the head. This indicates the prominence of black melanin over brown melanin. A similar trend was also observed in the thorax (FPKM 203, 42 and <1) and abdomen (185, 44 and <1). It hence appears that in *I. elegans* the formation of black melanin is prominent over brown melanin.

We were also able to identify a large number of patterning genes. We found seven regulatory genes in nine transcripts, namely *doublesex*, *abdominal-a*, *wingless*, *decapentapleic*, *bric-a-brac*, *ultrabithorax* and *distal-less* (Additional file 1: Table S12). Extensive pigmentation studies performed on *D. melanogaster* and other insects have reported a role of some of these regulatory elements in pigmentation patterning with strong links to sexual selection, sexual dimorphism and speciation in these more modern insect groups [46, 68, 69]. In the future, identification of pattering genes can help to answer questions related to sex-specific pigmentation in *I. elegans* and other odonate species that show genetic colour polymorphisms.

### Other important findings

#### Odorant-binding protein and receptors

We identified odorant-binding proteins (ejaculatory bulb-specific protein 3 and odorant-binding proteins 4), Olfactory Receptor (OR), Ionotropic Receptors (IRs) and Gustatory Receptors (GRs) that were expressed in all three tissues (Additional file 1: Table S13). This is of principal interest as odonates have until quite recently been thought to have poor olfaction and mainly communicate through visual signals. Recent behavioural and electrophysiological work, however, indicates that olfaction might be important also in odonates, at least in the context of foraging [70, 71]. Future studies on odonates should investigate if the odour and taste receptors that have been shown to be important in the detection of food, mates and oviposition sites in modern insects like *Drosophila* [72] are operating also in this very ancient insect group, which its long history of independent evolution from the well-investigated model insect systems.

#### Heat and cold shock proteins

We identified seven different types of heat shock proteins (HSP), three types of heat shock factors and two cold shock proteins in 35 transcripts. The HSP identified are HSP 10, HSP 70 (heat shock 70 kda protein 14, heat shock 70 kda protein 4l, heat shock 70 kda protein cognate 3, heat shock protein 70 kda protein cognate 5), HSP 75, HSP 60, HSP67b2, HSP90, HSPgp96, heat shock factor, heat shock factor 2-binding protein, heat shock factor binding and small HSP. The most expressed HSP in the head was HSP 70 (FPKM = 372) followed by HSP 90 (FPKM = 324), in the thorax small HSP (FPKM = 547) followed by HSP 70 (FPKM = 173) and in the abdomen HSP 70 (FPKM = 407) followed by smallHSP (FPKM = 239).

The cold shock proteins identified are cold shock domain-containing protein e1 and cold shock domain protein a. For detailed description about the expression and homology of heat and cold shock proteins refer Additional file 1: Table S14.

#### Transient receptor potential (TRP) channel

TRP channels are of ecological interest for research on thermal adaptation, as these insects are known to thermoregulate, in spite of being ectothermic animals [73, 74]. Moreover, some of the larger odonates (dragonflies) can even generate heat internally, through muscle movement and thermogenesis [75]. The ability to thermoregulate is likely to be under strong natural and sexual selection, with latitudinal gradients and phylogenetic inertia are likely to have jointly shaped the phenotypic traits underlying thermal plasticity and thermal niches [7]. Here, we identified eight different types of TRP in 39 transcripts. The different types of TRP identified are TRP channel, TRP cation channel subfamily A member 1-like (TRPA1 isoform g, TRPA1 isoform k, TRPA1 isoform i), TRP cation channel subfamily v member 6, TRP channel pyrexia, TRP cation channel protein painless-like, TRP cation channel cg34123-like, short TRP channel 5-like and TRP-gamma. In head TRP channelwas most expressed (FPKM = 98), followed by TRP cation channel subfamily A (FPKM = 28), whereas in thorax and abdomen TRP channel was most expressed (FPKM = 14 and FPKM = 40), followed by TRP cation channel cg34123 (FPKM = 13 and FPKM = 32) (Additional file 1: Table S15).

## Conclusions

The *de novo* transcriptome of *I. elegans* is the most complete transcriptome assembly of an odonate species to date and fills a major taxonomic gap. The annotated genes provide an important toolkit for future studies on colour, vision, olfaction and temperature sensitivity in this and other species. In particular, the data from this study will provide baseline knowledge for future studies investigating the molecular and genomic basis behind the evolution of colour polymorphism in Odonata, and the associated changes in vision, which may have facilitated phenotypic divergence in this ancient insect order. Moreover, the findings in this study should also facilitate future comparative genomic investigations between odonates and more modern insect groups, including model organisms like *Apis mellifera*, *Drosophila melanogaster* and *Tribolium castaneum*.

## Methods

### Data collection and sample preparation

One adult male *I. elegans* was collected from Alphen aan den Rijn in the Netherlands, on the 3^rd^ of August 2011. The individual was immediately euthanized in EtOH (<10 sec) upon capture. The head, thorax and abdomen were separately crushed and stored in RNA later and from each of the three tissue types RNA was extracted. The tissue was homogenized using a bullet blender. Total RNA was extracted using an RNeasy kit (Qiagen) using the standardized instructions from the manufacturer. An aliquot of the extracts was used to quantify RNA using a RNA nano chip. mRNA was extracted, fragmented, converted to cDNA and fitted with adapters using standard protocols at the LGTC (Leiden Genome Technology Center, Netherlands). The libraries were PCR amplified for 16 cycles (10 μl cDNA prep, 10 μl Phusion hot start buffer 7.5 mM MgCl2, 1 μl 10 mM dNTP’s, 1 μl P1, 1 μl P2, 10 μl DNA, 0.5 μl Phusion, 20 μl water, 1 μl USER; 30 min 37°C, 45 min 98°C, 10 min 98°C, 30 min 60°C for 15 cycles and 30 min 72°C). Sequencing was performed in November 2011 with an Illumina HiSeq 2000 at LGTC using paired-end reads with an insert size of 280 bp and an adapter length of 60 bp.

RNA sequence data has been deposited in the National Center for Biotechnology Information (NCBI) database under *Ischnura elegans* BioProject: PRJNA245854, which contains links and access to insect sampling data through the BioSample link: SAMN02741069 and the Sequence Read Archive: SRR1265958.

### Data processing and *de novo*transcriptome assembly

The raw sequencing reads were trimmed by removing adapter sequences. Low quality sequences with an average quality score of less than 20 were removed using Nesoni clip version 0.109. Subsequently, reads with a length of less than 24 bp were also discarded and the remaining reads were used for the assembly. The trimmed reads from head, thorax and abdomen were *de novo* assembled using Trinity version trinityrnaseq_r2012-06-08 [28, 29]. Trinity generates transcriptome assemblies from short read sequences using the de Bruijn graph algorithm. The parameters selected to run Trinity were all default parameters (kmer length = 25-mers) except min_kmer_cov which was set to 2.

### Assembly quality assessment

In order to assess the quality of the assembly, the Alignment Visualization and Quality Assessment application within Trinity software was used. This maps the reads back to the assembled transcripts using the bowtie aligner. The mapping results were visualized using Integrated Genomics Viewer version 2.3.2 (IGV) [30].

To improve the quality of the assembly, duplicates were removed and an internal quality check was performed. To remove duplicates from the assembly, clustering was performed using CD-HIT-EST at 95% sequence similarity [34]. The application genome coverage bed within BED Tools version 2.17.0 was used to calculate the read coverage at each base. The transcripts with a mean coverage per base of less than five were removed from the assembly, because of the increased likelihood that these had been misassembled. The assembled transcripts were also screened for repetitive elements and rRNA using RepeatMasker version 4.0.1 using the default mode [35]. RepeatMasker was run with rmblastn version 2.2.27+ on RepBase update 20130422 and RM database version 20130422. The sequence completeness of the assembly was estimated with CEGMA software [36] and TargetIdentifier [37]. CEGMA version 2.4.010312 was used to evaluate the completeness of a transcriptome assembly by estimating the presence and completeness of 248 ultra-conserved eukaryotic genes. It uses profile-hidden Markov model to ensure reliability of gene structure. Default parameters were used to run CEGMA. TargetIdentifier identifies the full-length transcripts using the BLASTX alignment as a guide to identify the protein coding regions and potential start and stop codons. The parameters that were used to run BLASTX are -v 1 -b 1 1E-5 on NCBI non-redundant protein database. Likely coding regions (Open Reading Frame) in the transcripts were identified using Transdecoder, which is an application within the Trinity software version trinityrnaseq_r2013_08_14.

### Functional annotation of transcripts

High quality transcripts were annotated with the BLAST2GO [38], a comprehensive suit designed for the functional annotation and analysis of gene and protein sequences. The sequence homology search was conducted with BLASTX against the NCBI non-redundant (nr) protein database version 13^th^ November 2013 using an e-value cutoff of 1E-5. The conserved motifs/domains were identified using InterProScan on the six possible translational frames of each transcript. The transcripts were functionally annotated according to the Gene Ontology nomenclature. InterProScan ID's were also mapped to GO terms and were merged with blast derived GO annotations in order to obtain one fully integrated annotation result. The GO annotations were further refined into biological processes, cellular components and molecular functional annotations. A GO_Slim reduction was performed on GO terms to obtain more precise GO definitions. Default settings were used to perform BLAST2GO, GO_Slim, GO Term enrichment and InterProScan analysis.

### Abundance estimation and differential expression

An abundance estimation of the transcriptome assembly was obtained with the RSEM version 1.2.7 [41], separately for the three sets of filtered reads from the head, thorax and abdomen. RSEM is a package used to estimate the gene and isoform expression levels from RNA sequence data. RSEM was run using the default parameters except the seed-length, which was set to 24, while calculating the expression. The relative measure of transcript abundance was TPM (Transcripts Per Million) and FPKM (Fragments Per Kilobase of transcript per Million mapped reads).

Differentially expressed transcripts were identified using edgeR Bioconductor [76]. EdgeR uses a negative binomial distribution method for differential expression analysis. We used edgeR through ‘Identification and analysis of differentially expressed genes and transcripts’ application with Trinity software versiontrinityrnaseq_r20140413 at default settings.

### Interspecific comparisons

The quality trimmed reads were mapped to the *D. melanogaster* transcriptome (downloaded on October 20^th^ 2013 from the Ensembl database [Ensembl:BDGP5]) [27]. In addition, we mapped the paired-end reads from *L. fulva* (downloaded from Baylor College of Medicine Human Genome Sequencing Center ftp site under the I5K project [BCM-HGSC:I5K]) [25] and the single end reads from *E. Hageni* (downloaded from NCBI [NCBI:SRR649536] submitted by BioProject number PRJNA185185 ID:185185) [26] to the *I. elegans* transcriptome. All mapping was performed using Bowtie2 with default parameters accompanied by Samtools for format conversions and for summarizing the mapping statistics [77, 78]. We considered only those transcripts as mapped to which more than three reads were aligned.

All the computations were performed on resources provided by SNIC through Uppsala Multidisciplinary Center for Advanced Computational Science (UPPNEX) under Project b2013227 [79].

## Electronic supplementary material

Additional file 1: Table S1: Summary of repeats identified in *I. elegans.* **Table S2.** Comparative assembly statistics before and after filtering. **Table S3.** List of 30 most expressed genes from *D. melanogaster* that expressed in *I. elegans.* **Table S4.** List of 30 most expressed genes from *I. elegans* that expressed in *E. hageni* and *L. fulva.* **Table S5.** List of 20 most expressed genes in head of *I. elegans.* **Table S6.** List of 20 most expressed genes in thorax of *I. elegans.* **Table S7.** List of 20 most expressed genes in abdomen of *I. elegans.* **Table S8.** List of 20 differential expressed genes in head of *I. elegans.* **Table S9.** List of 20 most differential expressed genes in thorax of *I. elegans.* **Table S10.** List of 20 most differentially expressed genes in abdomen of *I. elegans.* **Table S11.** Expression and similarity statistics of different type of opsins expressed in *I. elegans.* **Table S12.** Details of locus similarity and expression levels of pigmentation enzymes and regulatory elements of *I. elegans.* **Table S13.** Odorant-binding proteins, olfactory receptor, ionotropic receptors and gustatory receptors identified in *I. elegans* with their expression in three tissues. **Table S14.** Expression and similarity statistics of different type of Heat and cold shock proteins expressed in *I. elegans.* **Table S15.** Reports different types of TRPs identified in *I. elegans* with their expression in three tissues. (XLSX 50 KB)

Additional file 2: Figure S1: Comparison between filtered (final) and un-filtered (initial) transcriptome of *I.elegans.* **Figure S2.** Gene Ontology annotation for biological process category *I. elegans.* **Figure S3.** Gene Ontology annotation for Cellular Component category *I. elegans.* **Figure S4.** Gene Ontology annotation for Molecular Function category *I. elegans.* **Figure S5.** Venn diagram deciphering the distribution of *E. hageni* (blue) and *L. fulva* (yellow) transcripts expressed in *I. elegans*. **Figure S6.** Enriched GO term distribution observed in head of *I. elegans* after reducing to most specific GO terms. **Figure S7.** Enriched GO term distribution observed in thorax of *I. elegans.* **Figure S8.** Enriched GO term distribution observed in abdomen of *I. elegans* after reducing to most specific GO terms. (DOCX 863 KB)
