# Supplementary material for: De novo transcriptome of Ischnura elegans provides insights into sensory biology, colour and vision genes
Source: BMC Genomics. 2014 Sep 22;15(1):808. doi: 10.1186/1471-2164-15-808 (PMC4182773; doi:10.1186/1471-2164-15-808)
Supplement: Supplementary file 2 — Additional file 2: Figure S1: Comparison between filtered (final) and un-filtered (initial) transcriptome of I.elegans. Figure S2. Gene Ontology annotation for biological process category I. elegans. Figure S3. Gene Ontology annotation for Cellular Component category I. elegans. Figure S4. Gene Ontology annotation for Molecular Function category I. elegans. Figure S5. Venn diagram deciphering the distribution of E. hageni (blue) and L. fulva (yellow) transcripts expressed in I. elegans. Figure S6. Enriched GO term distribution observed in head of I. elegans after reducing to most specific GO terms. Figure S7. Enriched GO term distribution observed in thorax of I. elegans. Figure S8. Enriched GO term distribution observed in abdomen of I. elegans after reducing to most specific GO terms. (DOCX 863 KB) [file 12864_2014_6489_MOESM2_ESM.docx]

**Figure 1. Comparison between final (filtered) and initial (un-filtered) transcriptome of *I. elegans.*** x axis represents transcript length and y represents transcripts frequency. Filtering step proved important for obtaining quality transcripts as it not only removed the redundancy but also filtered shorter transcripts.


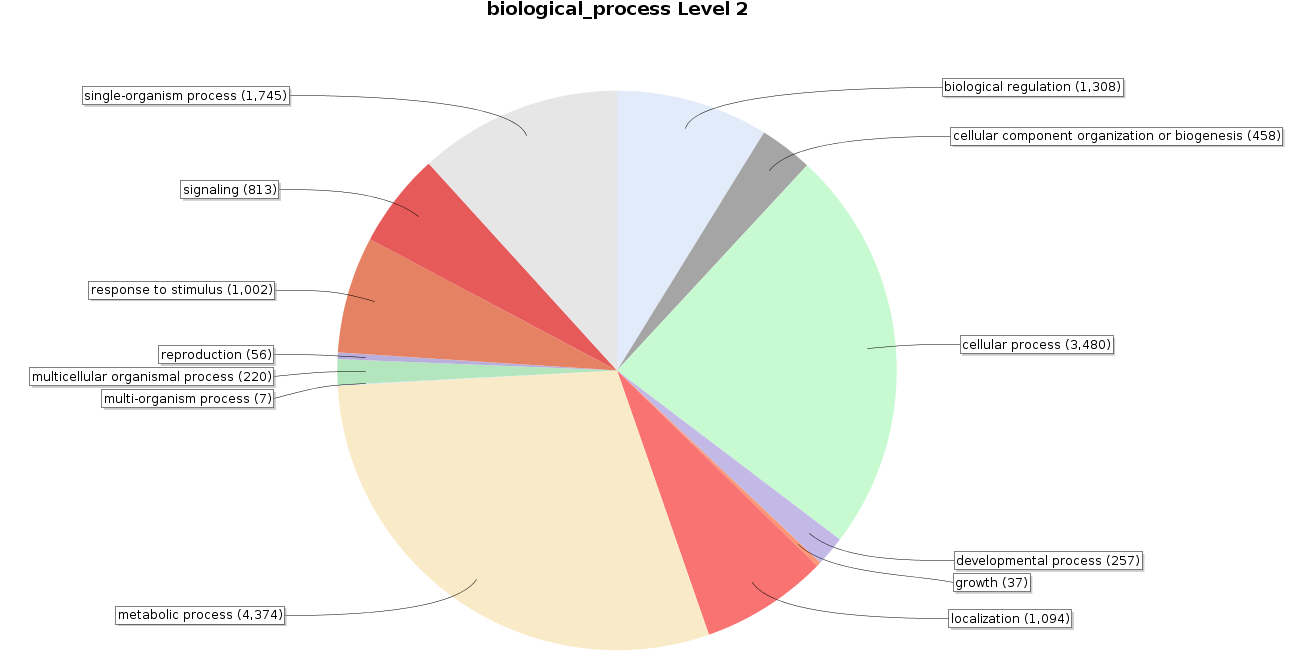


**Figure 2. Gene Ontology annotation for biological process category *I. elegans.*** The figure describes GO annotation (GOslim) at second level of ontology


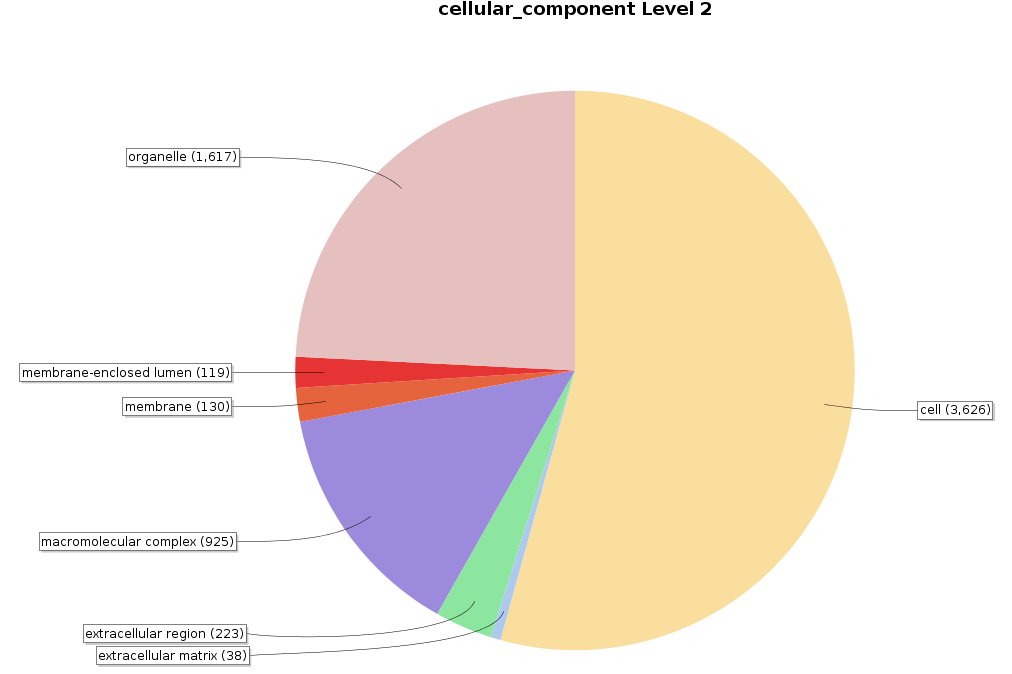


**Figure 3. Gene Ontology annotation for Cellular Component category *I. elegans.*** The figure describes GO annotation (GOslim) at second level of ontology.


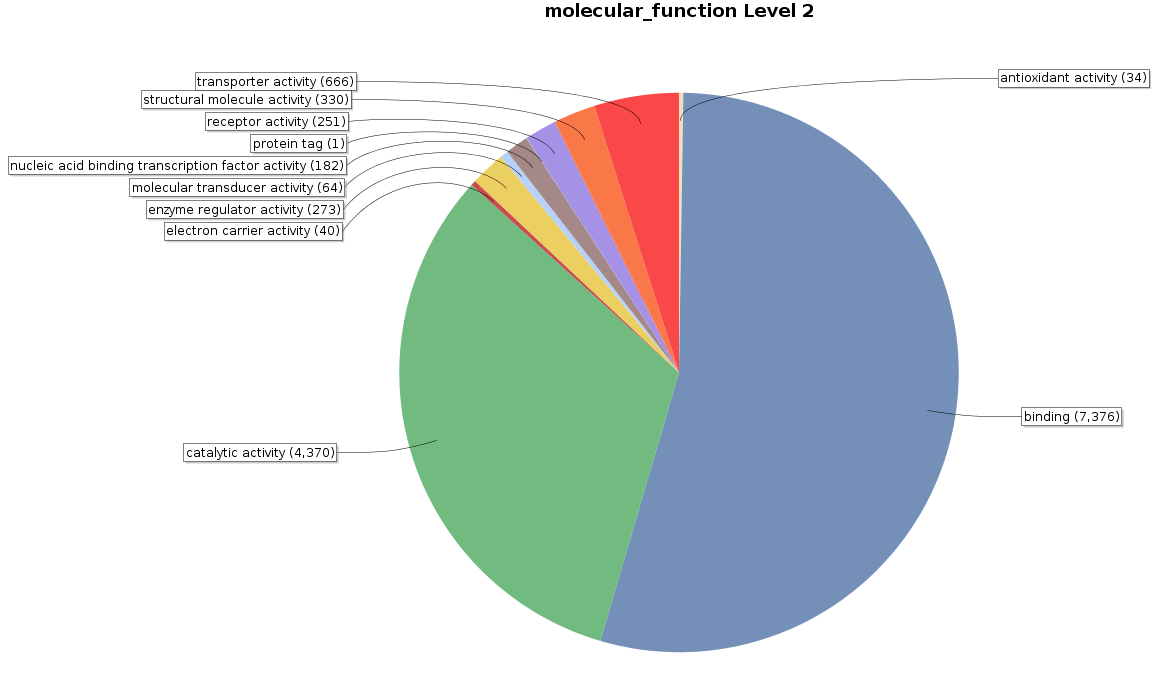


**Figure 4. Gene Ontology annotation for Molecular Function category *I. elegans****.* The figure describes GO annotation (GOslim) at second level of ontology.


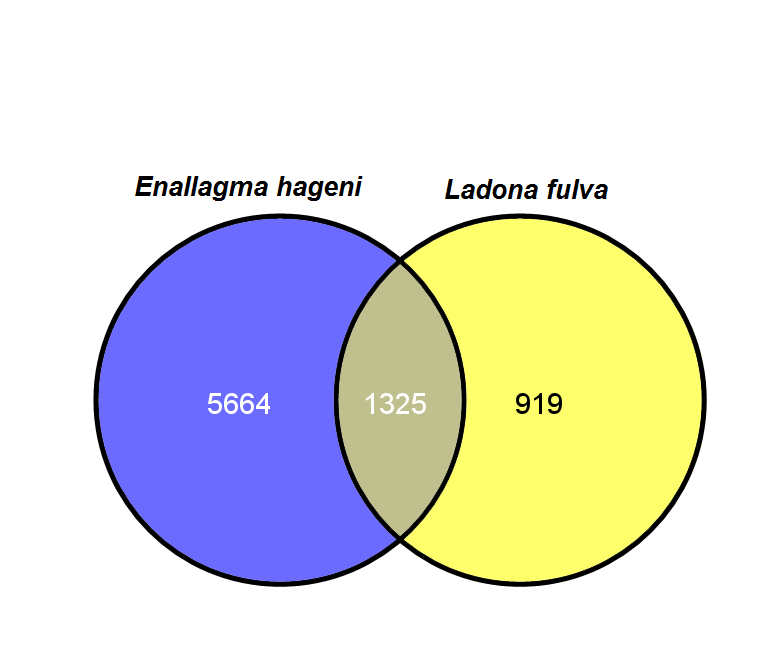


**Figure 5. Venn diagram deciphering the distribution of *E. hageni* (blue) and *L. fulva* (yellow) transcripts expressed in *I. elegans*.** A total of 6,989 *E. hageni and* 2,244 *L. fulva* transcripts showed expression in *I. elegans*. Among them 1,325 transcripts were expressed in both the species.


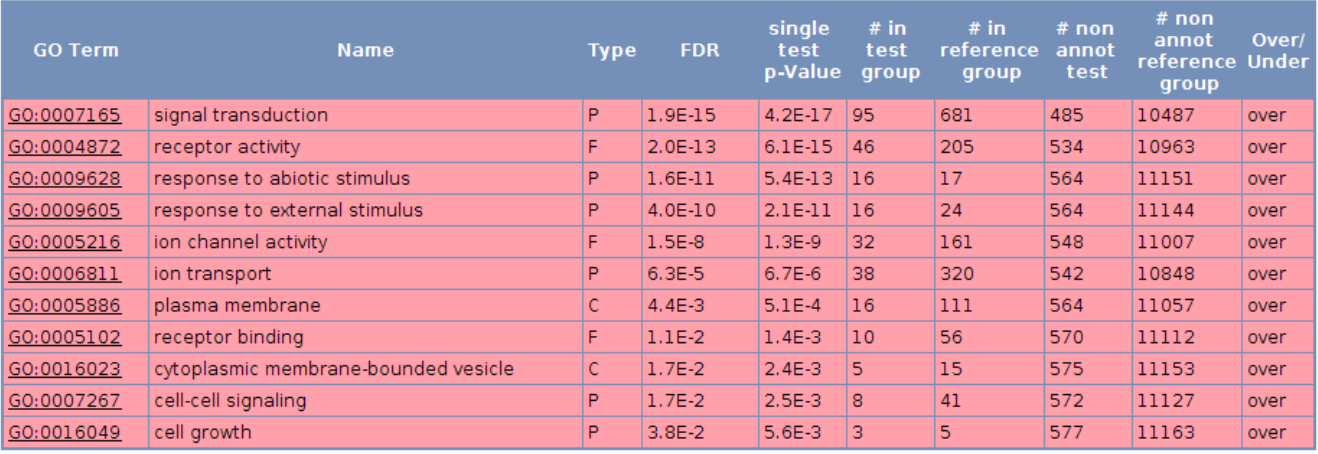


**Figure 6. Enriched GO term distribution observed in head of *I. elegans* after reducing to most specific GO terms.** The enrichment is performed on differentially expressed genes of head against transcriptome dataset.


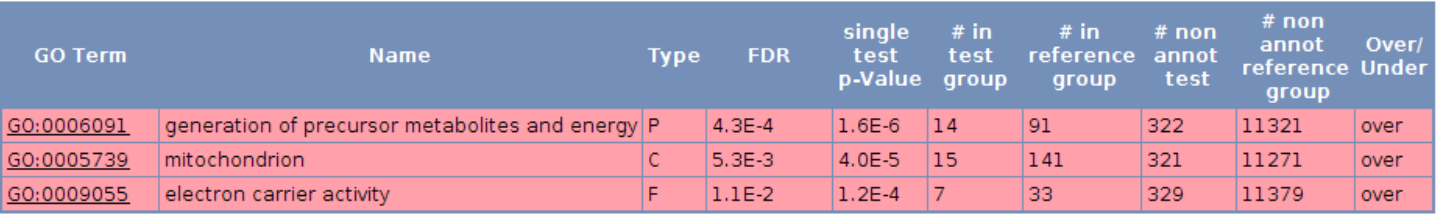


**Figure 7. Enriched GO term distribution observed in thorax of *I. elegans.*** The enrichment is performed on differentially expressed genes of thorax against transcriptome dataset.


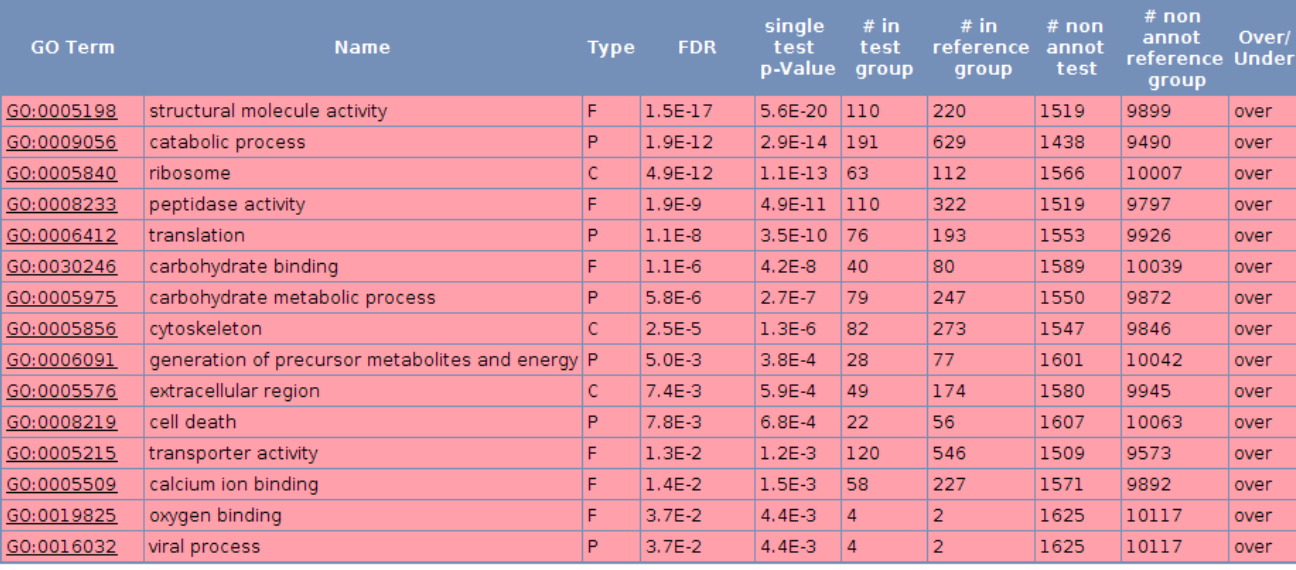


**Figure 8. Enriched GO term distribution observed in abdomen of *I. elegans* after reducing to most specific GO terms.** The enrichment is performed on differentially expressed genes of abdomen against transcriptome dataset.
